# Telehealth provider experience in reproductive endocrinology and infertility clinics during the COVID-19 pandemic and beyond

**DOI:** 10.1007/s10815-022-02549-2

**Published:** 2022-06-22

**Authors:** Elizabeth A. Dilday, Christopher R. Douglas, Zain A. Al-Safi

**Affiliations:** Department of Obstetrics and Gynecology, Division of Reproductive Endocrinology and Infertility, UCLA David Geffen School of Medicine, 10833 Le Conte Avenue, 27-139 CHS, Los Angeles, CA 90095-1740 USA

**Keywords:** Telehealth, Reproductive endocrinology, Fertility, COVID-19 pandemic

## Abstract

**Purpose:**

To assess telehealth services offered by reproductive endocrinology and infertility specialists and to gauge provider experiences with incorporating telehealth into their practices.

**Methods:**

A 16-question web-based survey on use of telehealth was distributed to Society for Assisted Reproductive Technology (SART) clinics and to Society for Reproductive Endocrinology and Infertility (SREI) members. Clinic demographic data, telehealth descriptive data, and provider satisfaction with use of telehealth were assessed. Results were collected via Survey Monkey.

**Results:**

A total of 1160 individuals (330 SART clinic contacts and 830 SREI members) were reached via email with an 18.6% (216) survey response rate. All respondents indicated that they offer telehealth visits. Several telehealth platforms were used, with Zoom (62.7%) and telehealth through the clinic’s electronic medical record platform (34.8%) being the most common. The majority of participants (87.0%) anticipate they will offer telehealth visits after the COVID-19 pandemic. Roughly two-thirds (64.4%) of respondents anticipate fewer telehealth visits after the pandemic because of logistics, cost, and patient/provider preference. Nearly all providers are either “very satisfied” (66.2%) or “somewhat satisfied” (31.0%) with telehealth overall.

**Conclusion:**

Telehealth enabled safe patient-provider interactions throughout the COVID-19 pandemic. While only one-third of survey respondents offered telehealth services before the pandemic, nearly all providers express satisfaction with telehealth and anticipate they will offer telehealth services henceforth.

## Introduction

Telehealth is broadly defined as the “use of electronic communication technologies to provide and support health care when distance separates the patient and health care professional” [1]. It employs video-teleconferencing and other tools to develop a communication network that enables physicians off site to offer patient consults, among other services [1].

The coronavirus disease 2019 (COVID-19) pandemic promoted and accelerated the implementation of telehealth [2]. Before the pandemic, telehealth facilitated access to care in specific scenarios: for evaluation of patients in rural areas, for diagnosis when physical examination may not be essential (e.g., diagnostic radiology), or for treatment of disease where outcomes can be remotely assessed (e.g., dermatology) [3]. Nowadays, telehealth use in specialized and urgent care is increasing more than ever [3]. It is now embedded in the daily practice of providers across the health care spectrum, including in reproductive medicine [4].

In the USA, provider ability to deliver fertility care and perform reproductive surgery was restricted with the pandemic onset. In March 2020, state and local governments as well as medical professional societies recommended the limitation of non-emergency medical care to prioritize health care resources for the care of patients diagnosed with COVID-19 [5]. This led to cancellation of elective assisted reproductive technology (ART) treatment cycles and near shutdown of in vitro fertilization (IVF) labs out of an abundance of caution [6]. Telehealth became prominent in the field of reproductive endocrinology and infertility (REI), just as it did in many other medical specialties and subspecialties, in an effort to improve access to care, sustain clinic operations, and evade treatment delays. Telehealth also reduces risk of exposure to vulnerable patients and protects the health care workforce [7].

There have been numerous studies on the use of telehealth in other specialties and treatment settings [3, 4, 7]. More studies have been published recently describing how different specialties used telemedicine to care for patients during the pandemic [3]. To date, no information about the use of telehealth in REI practices has been published, given its recent widespread adoption in this subspecialty. As a result, we set out to fill a gap in existing knowledge and gather information on the use of telehealth in the field of REI. The study objective was to assess telehealth services offered by Society for Assisted Reproductive Technology (SART) clinics and Society for Reproductive Endocrinology and Infertility (SREI) members and to elucidate provider experiences with incorporating telehealth into REI practices.

## Material and methods

The authors collaborated to develop a 16-question web-based survey on the use of telehealth (Table 1). The response formats were selected through Survey Monkey. The survey was first distributed to all 330 SART member clinic physicians through email. Contact email addresses were acquired from the “Find a Clinic” feature on the SART website. The survey opened on April 14, 2021. Three reminder emails were sent to clinics that had not completed the survey over a 2-week period. Then, to reach a broader audience in order to attain more generalizable results, the survey was sent to the SREI research committee for approval for its distribution to all SREI members. After authorization, the survey was distributed to all 830 SREI members through the SREI research committee on August 20, 2021. One reminder email was sent to all SREI members on September 13, 2021, and the survey closed the following week.

**Table 1:**
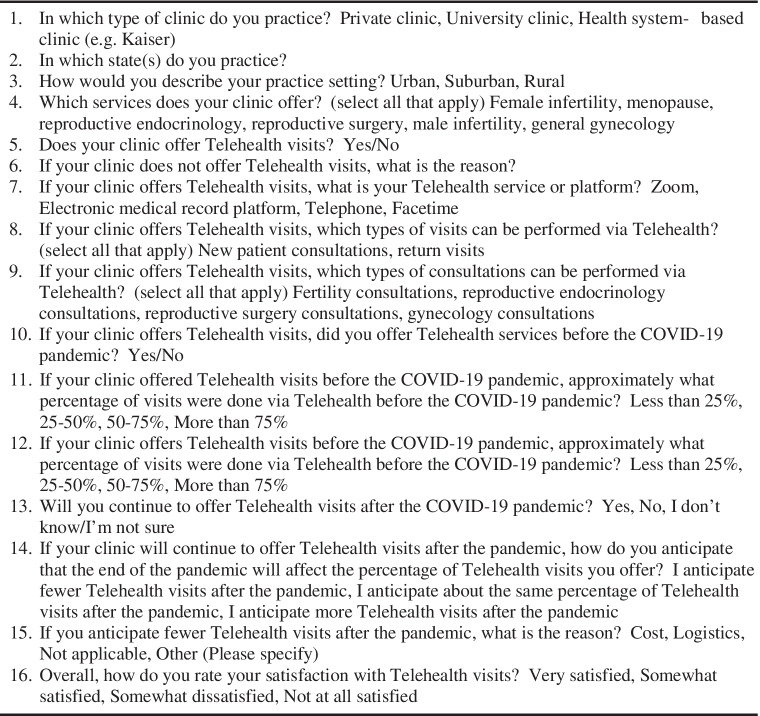
Survey questions

Clinic demographic data, telehealth descriptive data, and provider satisfaction with use of telehealth were assessed. Results were collected and analyzed via Survey Monkey.

## Results

A total of 216 individuals responded to the survey out of 1160 who were reached, with a response rate of 18.6%. 55.8% (120) of respondents were from private clinics, 37.7% (81) were from university-based clinics, and 6.5% (14) were from health system-based clinics (e.g., Kaiser Permanente Medical Group).

The respondents represented 36 unique states plus the District of Columbia, with 13.3% (28) from California, 8.1% (17) from Illinois, and 8.1% (17) from New York. 51.7% (109) of respondents described their practice setting as urban and 47.9% (101) as suburban. Only 1 respondent (0.5%) described their practice setting as rural, but some commented that their urban practice included a large rural referral base and others reported having offices in multiple locations.

Nearly all (98.6%) individuals surveyed provided female infertility care, whereas 93.1% offered reproductive endocrinology care. 80.6% of providers performed reproductive surgery and 71.8% provided male infertility care. A minority of individuals surveyed offered menopause management (32.4%) and general gynecology care (20.8%).

All survey respondents (100%) offered telehealth visits during the COVID-19 pandemic. The majority of these individuals (62.7%, 101) used Zoom, while roughly one-third (34.8%, 56) used telehealth through the existing electronic medical platform. Another 24.2% (39) of individuals reported using the telephone and 1.9% (3) used FaceTime. In the comments section of the survey, other platforms used included Doximity (33), Microsoft Teams (8), Ring Central (7), Google (3), GoToMeeting, Veracity, WebEx, SecurreVideo, VSEE, AmWell, and BlueJeans.

**Fig. 1:**
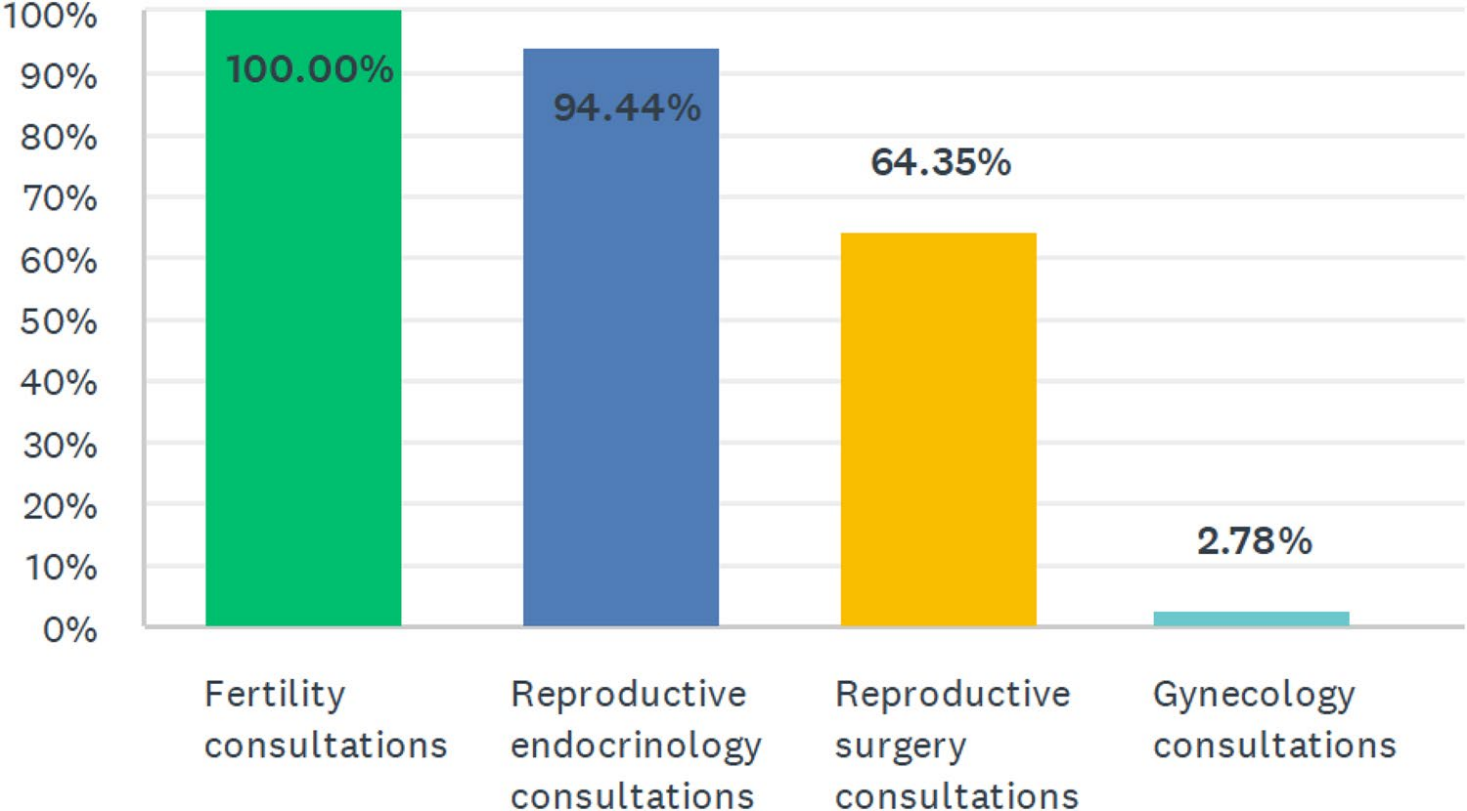
Types of visits offered via telehealth at REI clinics surveyed

Nearly all individuals surveyed used telehealth for new patient consultations (96.7%, 208) and return visits alike (98.1%, 211). All clinics (100%) offered fertility consultations via telehealth (Fig. 1). Nearly all (94.4%, 204) offered telehealth reproductive endocrinology consultations, two-thirds (64.4%, 139) offered reproductive surgery consultations, and a few (2.8%, 6) offered gynecology consultations.

Only 35.7% (77) offered telehealth services before the pandemic. Of those who provided telehealth services before the pandemic, nearly all stated that less than 25% of visits were done via telehealth before COVID-19. During the COVID-19 pandemic, on the other hand, more than 75% of visits were done via telehealth for more than half of those clinics (58.8%).

The survey queried provider predictions for telehealth henceforth. 87.0% of respondents (188) stated that they will continue to offer telehealth visits after the COVID-19 pandemic, while 12.5% (27) were unsure about future use. Two-thirds (64.4%) anticipate fewer telehealth visits after the pandemic, 29.6% anticipate about the same percentage of visits after the pandemic, and 5.1% anticipate even more telehealth visits after the pandemic.

The survey respondents who anticipate fewer telehealth visits after the pandemic offered various explanations. Most cited logistics, cost implications, lack of insurance coverage, patient preference, and provider preference as reasons that telehealth may decline in use after the pandemic.

**Fig. 2:**
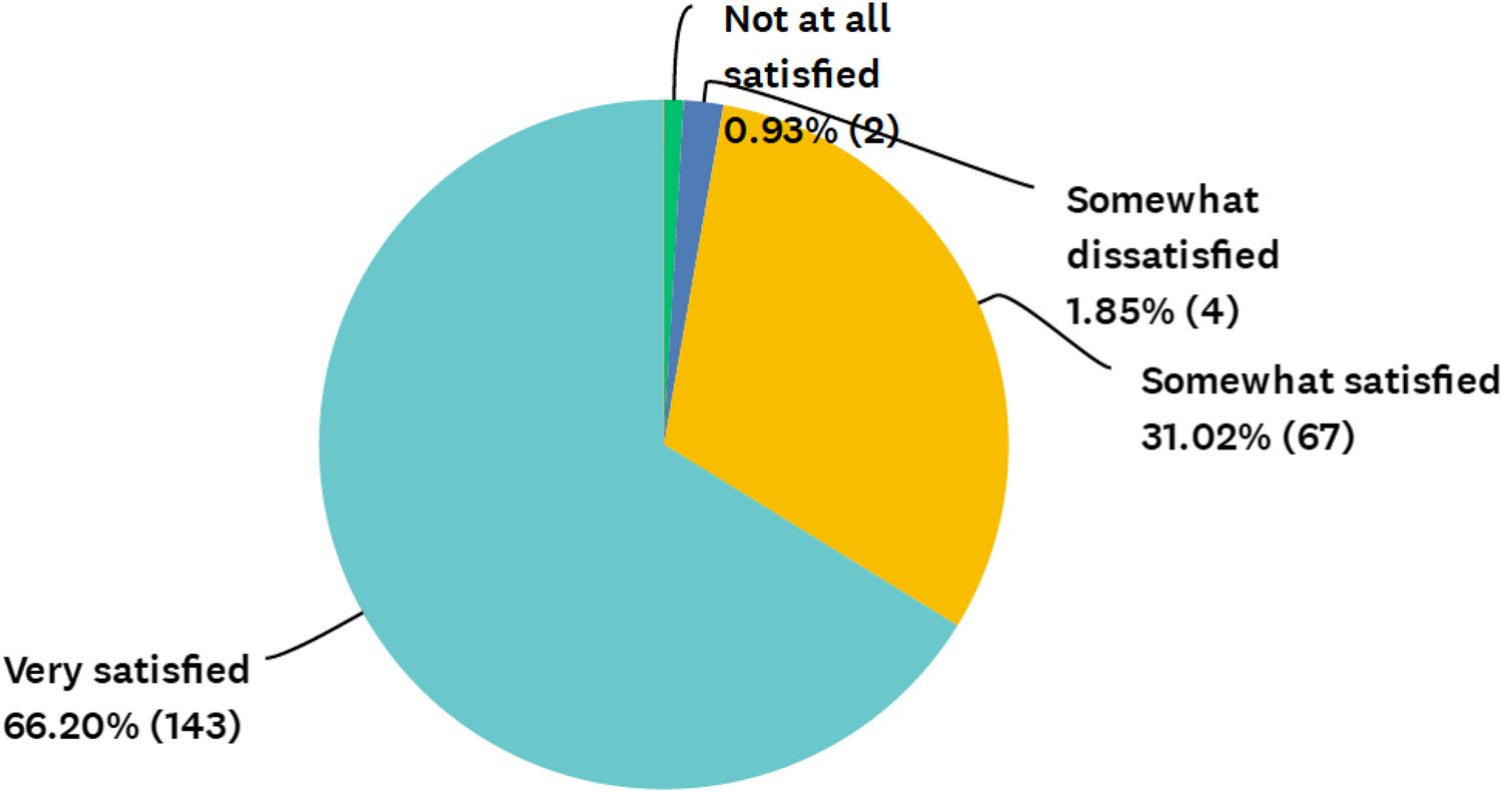
Provider satisfaction with telehealth visits

Most providers express great satisfaction with telehealth. 66.2% (143) stated they are very satisfied with telehealth visits and 31.0% (67) are somewhat satisfied (Fig. 2). A small minority of respondents stated that they are somewhat dissatisfied (1.85%) or not at all satisfied (< 1%) with telehealth services.

## Discussion

The swift incorporation of telehealth for all individuals surveyed is one of many transformations that the field of reproductive medicine has seen with the pandemic [6]. Based on our results, there has been a shift in telehealth use: From one-third of individuals using telehealth relatively infrequently before the pandemic to all respondents using it during the pandemic, with more than half conducting the majority of visits in this fashion. Access to care that telehealth enabled was critical as pandemic-related uncertainty, social distancing mandates, and restrictions in everyday functions made the prospect of pregnancy increasingly complicated for patients [5].

The survey demonstrates widespread telehealth use in all REI practice settings: private, university-based, and health care system-based practices, and in both urban and suburban locations. Telehealth is used for new and return visits alike and for a variety of chief complaints (infertility, reproductive endocrinology, and reproductive surgery). The survey shows telehealth’s lasting impact on the practice of REI, as most providers express great satisfaction and anticipate telehealth use after the pandemic.

Telehealth utilization rates in our survey are consistent with those reported for other specialties. Reeves et al. explained that remote monitoring, access to multi-provider video visits, and virtual translators enabled some clinics to adopt a 100% virtual approach [4]. A review by Hincapie et al. reported that various hospitals in the USA saw a decrease of more than 80% of in-person visits, while ambulatory practices of diverse specialties reported virtual migration of between 60 and 95% of their usual practice [2, 8–11]. Similar to our assessment, all studies in the review have positively evaluated the experience and practicality of telemedicine [2]. While we aimed to gauge provider plans for telehealth following the pandemic, most studies did not comment specifically on potential for increased use in the future or make predictions for use after the pandemic [2, 4].

While telehealth has remained one of the medical community’s biggest allies during the pandemic by keeping patients safe through social distancing and maintaining self-quarantine [2, 3], some providers surveyed voiced frustrations. One concern was that telehealth may provide a lower quality of care in a field that routinely uses transvaginal ultrasound. The survey respondents who anticipate fewer telehealth visits after the pandemic offered justifications: concerns about insurance coverage and beliefs that in-person care will often be patient preference and even provider preference. However, a recent survey of patients undergoing new REI visits found that 92.5% of patients would use telemedicine services again and were satisfied with telehealth, and 82.5% stated that telehealth improved access to care [12]. In a separate study, a follow-up questionnaire distributed to fertility patients in January 2021 (comparing responses from April 2020) revealed that the top preference for consults was a combination of telemedicine and in-person consults (combined modalities with majority via telemedicine video/telephone (42.3%), or combined with majority in person (32.1%)) [13]. These results show that fertility care via telemedicine is satisfactory to patients and has become more conventional with time.

The strengths of this study are its reach of a wide audience across the subspecialty in many practice settings. It is the first survey of its kind to assess provider experiences with telehealth in REI practices and it offers an unprecedented opportunity to establish best practices.

Limitations include possible temporal bias, as opinions could have evolved in the year between pandemic onset and survey distribution. The response rate of nearly 20% may limit its interpretation. The survey is also subject to non-response bias, as those choosing to participate may feel more passionately about telehealth than the average provider does. We do not have information that illuminates differences and similarities between survey responders and non-responders. The survey could not determine whether a specific telehealth platform influenced one’s overall experience. Finally, while the email to SREI members explained that some of our colleagues had already responded with the first distribution of the survey to SART clinic physicians, there could have been potential for responder overlap.

The survey inspires us to ponder the future of REI clinical practice, as many changes motivated by increasing demand for services and limited supply of providers are on the horizon to promote efficient workflow. Hart et al. explain that remote urine-based hormonal assays still need thorough investigation before implementation, but hold promise to reduce inconvenience of serum tests, frequency of appointments, and requirement for costly skilled personnel for blood collection [14]. Remote testing also applies to use of self-operated home endovaginal sonography, which offers advantages of greater flexibility for patients and partners, less loss of income to attend appointments during working hours, and a more environmentally friendly approach due to reduced travel [15]. The adoption of at-home urinary hormonal assessment and remote ultrasound fit into the broader application of telehealth to link clinics and patients at home [14]. To further improve efficiency of clinical operations, telehealth can reduce the amount of travel for the provider to cover multiple clinic locations when all visits can be performed remotely from one location, allowing more patients to be seen in one day, which could help meet increasing demands. Telehealth is therefore one of the many changes to fertility care that has the potential to improve effectiveness of health care delivery.

Future directions include ongoing assessment of the patient and provider experience with telehealth in REI practices so that telehealth can be gratifying, efficient, and effective for providers and patients alike. It would be interesting to evaluate differences in provider preferences between televideo and telephone, given that telephone is used frequently in fertility clinics to communicate treatment plans and updates, and was a mode of telehealth used often among this survey’s respondents. Additional research can focus on improving telemedicine access, as the patients at highest risk of contracting COVID-19 potentially have the most to gain from telemedicine during the pandemic [7].

## Conclusions

While relatively few clinics offered telehealth services before COVID-19, the majority of participants in this survey anticipate that they will continue to offer telehealth after the pandemic, with most expressing great satisfaction. Future research would assess patient experience with telehealth, and on ways to overcome logistical issues to widen use of this important health care tool.
